# The Network of Inflammatory Mechanisms in Lupus Nephritis

**DOI:** 10.3389/fmed.2020.591724

**Published:** 2020-11-06

**Authors:** Yuji Nozaki

**Affiliations:** Department of Hematology and Rheumatology, Faculty of Medicine, Kindai University, Osaka, Japan

**Keywords:** TIM-1, KIM-1, nephritis, cytokines, immune response, kidney disease

## Abstract

Several signaling pathways are involved in the progression of kidney disease in humans and in animal models, and kidney disease is usually due to the sustained activation of these pathways. Some of the best understood pathways are specific proinflammatory cytokine and protein kinase pathways (e.g., protein kinase C and mitogen-activated kinase pathways, which cause cell proliferation and fibrosis and are associated with angiotensin II) and transforming growth factor-beta (TGF-β) signaling pathways (e.g., the TGF-β signaling pathway, which leads to increased fibrosis and kidney scarring. It is thus necessary to continue to advance our knowledge of the pathogenesis and molecular biology of kidney disease and to develop new treatments. This review provides an update of important findings about kidney diseases (including diabetic nephropathy, lupus nephritis, and vasculitis, i.e., vasculitis with antineutrophilic cytoplasmic antibodies). New disease targets, potential pathological pathways, and promising therapeutic approaches from basic science to clinical practice are presented, and the blocking of JAK/STAT and TIM-1/TIM-4 signaling pathways as potential novel therapeutic agents in lupus nephritis is discussed.

## Introduction

As a leading autoimmune disease, systemic lupus erythematosus (SLE) is a chronic inflammatory disease that affects multiple organs. SLE involves activations of dendritic cells (DCs), macrophages, and lymphocytes, which together lead to the production of high-affinity autoantibodies and immune complex formation. The pathogenesis of SLE remains unclear despite extensive clinical and animal studies. Various genes (1) and environmental factors including viral infections, hormones (2, 3), and ultraviolet light are thought to exacerbate SLE. An abnormal production and imbalance of T helper (Th) lymphocyte cytokines was demonstrated to be involved in the development of autoimmune diseases (4), and Th1 cytokines such as interleukin (IL)-2 and -12 and interferon-gamma (IFN-γ) and Th2 cytokines (e.g., IL-4, IL-5, IL-10, and IL-13) are also implicated in the pathogenesis of SLE. The inhibition of these cytokines is a key factor in the development of NZB/WF1 mice, which develop severe lupus-like phenotypes that resemble human SLE (5).

Th17 lymphocytes are a subset of Th cells with an important role in autoimmunity. These lymphocytes are derived from naïve CD4^+^ T cells and are characterized by the expression of the transcription factor RORγT (retinoic-acid-receptor-related orphan nuclear receptor gamma) (6). Once stimulated by various cytokines, including IL-23 (7), Th17 lymphocytes secrete cytokines such as IL-17 family members, IL-21, IL-22, tumor necrosis factor (TNF)-α, and IL-6 (6). Compared to healthy controls, individuals with SLE exhibited increased numbers of Th17 cells and IL-23 in their serum (8). Chen et al. observed that the frequency of circulating Th17 cells and the serum levels of IL-17 and IL-23 were higher in patients with loop nephritis compared to controls (9).

Th17 lymphocytes' potent pro-inflammatory effect has been shown to be due to the induction of vascular inflammation and the recruitment of leukocytes, and this is suspected to contribute to several pathological pathways in SLE, including the B-cell activation and autoantibody production observed in SLE (10). The imbalance of cytokines in SLE may be part of a core process of pathogenicity, or it may be a secondary marker of the dysregulation of immune pathways such as those involving Th1-Th2 and Th17-Treg cells (11, 12). IL-6 signaling *via* receptors (IL-6Rs) on activated B cells induces dimerization with the transmembrane protein gp130 and the activation of the receptor-associated Janus kinase (JAK) tyrosine kinases JAK1 and JAK2. This is the most important role of IL-6, as it is involved in multiple autoimmune diseases and contributes directly to the survival of plasma cells in the bone marrow niche (13).

Effector T cells also recognize autoantigens that are present in the kidneys as implanted or endogenous antigens (14–18), and fewer CD4^+^ and CD8^+^ cells are recruited to the glomerulus and stroma. The members of the T-cell immunoglobulin mucin-domain (TIM) family encode a protein that has an IgV-like domain and a mucin domain (19), and the three human TIM genes most similar to those in mice are TIM-1, TIM-3, and TIM-4. The roles of TIM proteins in T-cell differentiation, effector function, autoimmunity, and allergy are becoming clear (20), and it was demonstrated that TIM-1 is expressed on activated T cells (21). Another study suggested that TIM-1 on T cells acts as a costimulatory molecule to enhance cell proliferation and cytokine production and to mediate the loss of tolerance (22).

Chemokines and adhesion molecules are reduced by TIM-1 antibodies (18). In intracellular adhesion molecule-1 (ICAM-1) knockout mice treated with TIM-1 antibody, the renal and spleen mRNA expressions of the Th1 chemokines CXCL9 and CXCL10 were reduced and ICAM-1 mediated the recruitment of leukocytes in glomerulonephritis (23). A promising next research task would be to target inflammatory cytokines *via* a blockade of the JAK-signaling transducer and transcriptional activator (STAT) and TIM-1 signaling pathways, in order to better target the development and survival of autoreactive pathogenic plasma cells during the early stages of SLE.

In this review, new therapeutic targets for lupus nephritis, potential pathologies and promising therapeutic approaches to the JAK-STAT and TIM-1-TIM-4 signaling pathways from basic science to clinical practice are presented.

### Mechanisms Downstream of the JAK-STAT Pathway

Several signaling pathways are known to be involved in the progression of renal disease in both humans and animal models, and the progression is usually due to a sustained cytokine and JAK-STAT activation of these pathways (24). The JAK-STAT pathway is downstream of the type I and II cytokine receptors. As part of a major signaling cascade, JAK is an effective therapeutic target for a variety of cytokine-driven autoimmune and inflammatory diseases (25, 26). A cytosolic tyrosine kinase, JAK has been demonstrated to be an effective therapeutic target for a wide range of cell-surface receptors, and members of the cytokine receptor common gamma (cg) chain family in particular are involved in signaling (27).

**Figure 1:**
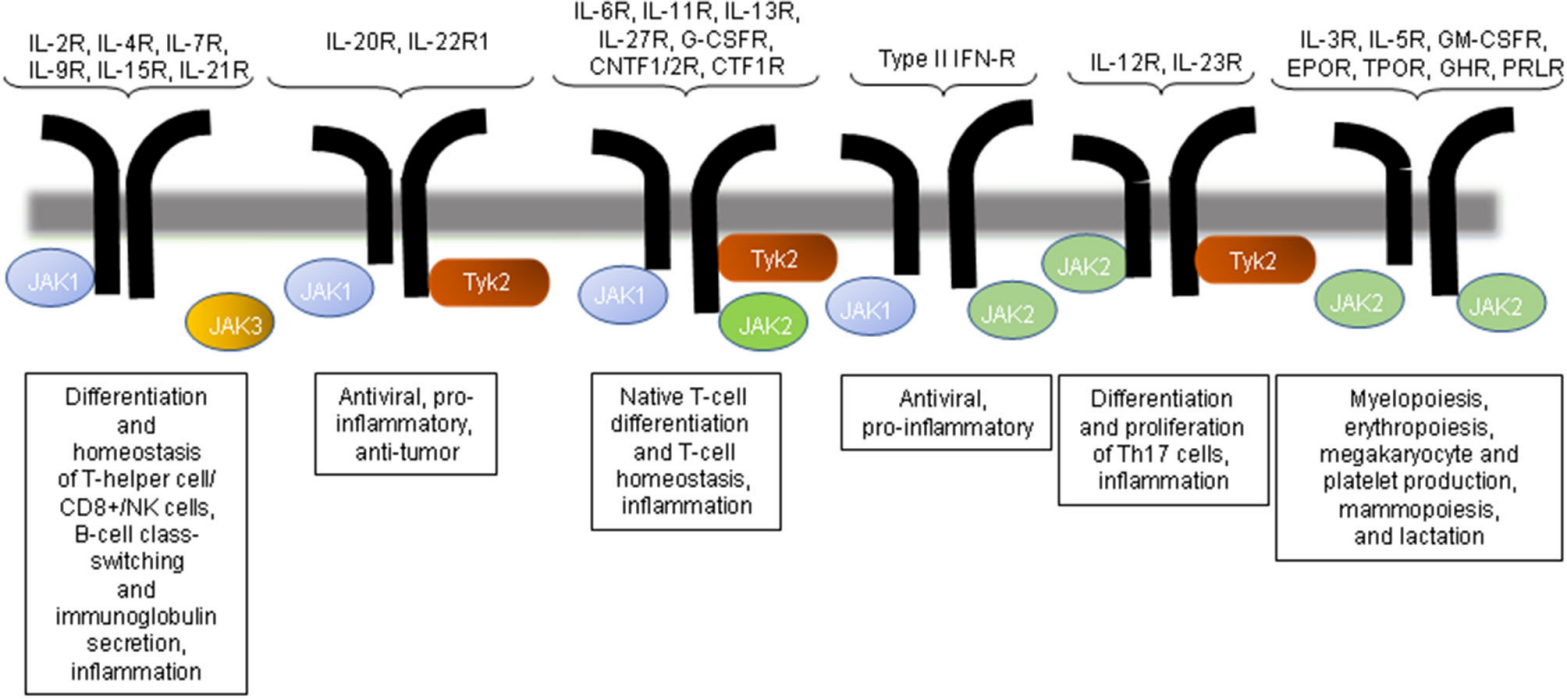
JAK inhibition and immune regulation by the JAK pathway. The inhibition of JAK3 would affect the signaling mediated by only the common gamma chain-associated cytokine receptors (IL-2R, IL-4R, IL-9R, and IL-21R) and regulate T-cell, NK cell, and B-cell function. JAK2 or Tyk2 inhibition would influence multiple cytokine receptor signaling pathways. CNTFR, ciliary neurotrophic factor receptor; EPO, erythropoietin; GH, growth hormone; GM-CSF, granulocyte macrophage colony-stimulating factor; IFN, interferon; IL, interleukin; NK, natural killer; PRL, prolactin receptor; TPO, thrombopoietin.

There are four mammalian JAKs: JAK1, JAK2, JAK3, and tyrosine kinase 2 (Tyk2). The activation of JAKs occurs *via* ligand-receptor interactions and results in the phosphorylation of the cytokine receptor; the signaling occurs *via* the generation of docking sites for signaling proteins known as STATs (19). JAKs catalyze the phosphorylation of STATs and promote STAT dimerization and nuclear transport, thereby regulating gene expression and transcription (28, 29). The JAK proteins are structurally related but different in their activation and their downstream effects; their high specificity is thus expected (Figure 1).

JAK1 is a receptor (IFN-α/β, IFN-γ, and IL-10) and γc, which is activated by ligands that bind to receptors (IL-2, IL-4, IL-7, IL-9, IL-15, and IL-21). JAK2 is activated primarily by the thrombopoietin receptor, IL-3, granulocyte-macrophage-colony stimulating factor (GM-CSF), and IFN-γ. Findings obtained with mouse models of SLE have repeatedly demonstrated the importance of IL-6 in promoting disease expression in SLE (30–32). Tyk2 mediates the signaling induced primarily by IL-12.

Although most of the JAKs are ubiquitously expressed, the expression of JAK3 appears to be restricted mainly to the hematopoietic system and vascular myocytes. JAK3 has an important role in lymphocyte development and function. JAK3 differs from the ubiquitous expression of the other JAK subtypes: it has a restricted tissue distribution, resides primarily on hematopoietic cells, and is associated with cg chains (33). The importance of the JAK3 signaling pathway was highlighted by the findings that mice and humans with genetic deletions or mutations in either the cg subunit or JAK3 develop defects in lymphocyte development, which result in a severe combined-immunodeficiency syndrome phenotype (34).

### The Blocking of the JAK-STAT Pathway as a Therapeutic Target

Since the JAK-STAT pathway has a major activating role in a variety of disease processes, concerted efforts have been made to develop specific inhibitors of this pathway. Inhibitors of protein kinases are relatively easy to identify, and the development of JAK inhibitors has received the most attention. The following three JAK inhibitors have been approved by the U.S. Food and Drug Administration (FDA) for clinical use.

Ruxolitinib (Jakafi®, from Incyte) is a potent inhibitor of both JAK1 and JAK2 and was FDA-approved in late 2011 for the treatment of polycythemia vera and myelofibrosis (35). In late 2012, the FDA approved tofacitinib (Xeljanz®, from Pfizer), which was initially designed as a specific inhibitor of JAK1 and JAK3 kinases; tofacitinib has also been administered as an immunosuppressant for the treatment of transplant patients and individuals with autoimmune diseases (36). The JAK2 inhibitor baricitinib (Olumiant, from Eli Lilly) was FDA-approved in June 2018 for the treatment of moderately to severely active rheumatoid arthritis (RA).

Several other JAK inhibitors have been developed as immunosuppressive agents for RA and other autoimmune diseases; e.g., upadacitinib (a JAK1 inhibitor) and filgotinib (a JAK1 inhibitor) were demonstrated to be effective as a treatment of RA (37, 38). Given the effects of JAK-STAT activation on cytokines and chemokines and the specific roles of inflammation in the promotion of progressive renal injury, it is not surprising that JAK-STAT activation is involved in the pathogenesis of both renal disease and acute kidney injury. The JAK-STAT pathway has been studied extensively, and due to its potent immunomodulatory function, the JAK-STAT pathway and its components are promising candidates for immunological interventions for disease control.

Indeed, JAK inhibitor clinical trials have been conducted for a variety of diseases including chronic kidney disease, RA, inflammatory bowel disease, atopic dermatitis, and psoriasis (39–41). There is significant interest in JAK-STAT as a therapeutic target for autoimmune nephritis in particular; the activation of JAK triggers the phosphorylation of IL-6R and gp130, followed by various secondary messengers including STAT3, mitogen-activated protein kinases (MAPKs), and Akt, all of which provide growth and proliferation signals and the activation of transcription factors (42).

Cytokines are glycated proteins with immunomodulatory functions that have important functions in infection and inflammation. Representative cytokines are members of the IL-6 family (which consists of IL-6, IL-11, IL-27, oncostatin M, cardiotrophin-1, and neuropoetin) (43). These cytokines are homo- or heteroduplexes of the signaling β-receptor gp130, which is expressed on ubiquitin. They are characterized by their quantified biological effects. A further transduction of signaling is carried out by the JAK/STAT, MAPK, and phosphatidylinositol-3-kinase (PI3K) pathways (44). Genetic excision or polymorphisms of key suppressors of JAK-STAT signaling, such as suppressors of cytokine signaling, have been implicated in elevated serum IL-6 levels and in the risk of SLE development in humans (45, 46).

JAKs also play an important role in transmitting signals from IL-6Rs, and IL-6 is involved in both SLE and the maintenance of a pool of potentially autoreactive plasma cells. The blockade of JAK signaling with selective and potent JAK2 inhibitors may therefore weaken the supportive effect of IL-6 on the maintenance of autoreactive plasma cells in SLE. Targeting the cytokine/growth factor pathway—which is important for plasma cell generation and the development of SLE—has been supported by several studies targeting the IL-6 pathway and receptors for the treatment of SLE (47); however, targeting IL-6 and IFN-γ failed to produce significant renal effects in either case.

The first *in vivo* study of the therapeutic use of the JAK/STAT pathway in lupus was performed in 2010 by Wang et al. (48). In that study, mice treated with the tyrosine kinase inhibitor AG-490 showed more inflammation (i.e., glomerulonephritis, interstitial nephritis, vasculitis, and even extra-renal features of the salivary glands as an extra-renal feature) than mice treated with the vehicle (inflammation). The inhibition of chemokines, IFN-γ, and major histocompatibility complex class II molecules on the surface of renal cells was observed. The AG-490 treatment also reduced the levels of blood urea nitrogen (BUN), serum creatinine, and proteinuria, and it reduced the depositions of IgG and C3 in glomerular cells. The study's immunohistochemical examination revealed a reduced expression of STAT1 in glomerular cells, tubular cells, and interstitial cells of the mice.

The effects of a selective JAK2 inhibitor (CEP-33779) on mice with lupus nephritis (LN) were assessed in a pivotal study conducted by Lu et al. (49). CEP-33779 protected MRL/lpr mice from the development of renal damage and ameliorated established disease in the mice, as well as in NZB/WF1 mice. In mice with pre-existing conditions, CEP-33779 resulted in increased survival, decreased proteinuria, the resolution of histological features of renal disease, and a decreased level of pSTAT3. Interestingly, CEP-33779 also reduced the levels of long-lived plasma cells in the spleen (at all doses) and in the bone marrow (at the highest dose). This effect may have therapeutic implications in human LN, given that long-lived plasma cells are involved in the production of antibodies. Conversely, treatment with CEP-33779 did not affect the levels of spleen short-lived plasma cells, which may be associated with a reduction in immunosuppression-related side effects (i.e., infections) and potentially associated with a better response to the vaccine (38, 50).

A specific blockade of JAK2 may also contribute to the treatment of SLE pathology, including arthritis and dermatitis. Multiple cytokines (IL-6, IL-12, and α/β-type IFNs) are suspected to have important roles in the initiation, progression, and development of SLE (51–54). These three cytokines are signaled through receptors regulated by JAK kinases. IL-6 signaling *via* IL-6R on activated B cells induces dimerization with gp130 and the activation of the receptor-associated JAK tyrosine kinases JAK1 and JAK2. This is the most important function of IL-6, since IL-6 is involved in multiple autoimmune diseases and contributes directly to the survival of plasma cells in the bone marrow niche (13).

In addition, multiple studies using mouse models of SLE have repeatedly demonstrated the importance of IL-6 in promoting disease expression in SLE (30, 31, 33, 52, 53). As noted earlier, the activation of JAK causes the phosphorylations of IL-6R and gp130, followed by growth and proliferation signals. JAK activates secondary messengers and transcription factors (e.g., STAT3, MAPK, and Akt) (43). Targeting the IL-6 pathway and receptors is currently being tested for the treatment of SLE (43, 55, 56).

Based on experimental and preclinical data, the oral selective JAK1 and JAK2 inhibitor baricitinib (which has been approved for the treatment of RA) was recently studied in 314 patients with epidural SLE, an epidural disease primarily involving the skin and joints, in a randomized, 24-week, placebo-controlled phase II trial (57). The patients were randomized 1:1:1 to two doses of baricitinib (4 mg or 2 mg/day) or placebo. The percentage of patients who achieved the resolution of arthritis or skin lesions was significantly higher in the 4-mg baricitinib group compared to the placebo group. Among the patients who received baricitinib, the SLE Disease Activity Index 2000 score at week 24 had decreased by >4 points and the British Isles Lupus Assessment Group A or B disease activity score did not worsen, and the Physician's Global Assessment.

In the trial (57), the percentage of patients whose disease activity and SLE Responder index-4 (as defined) did not worsen (64%) was also significantly higher than that in the placebo group (48%). The improvement in the number of tender joints was significantly higher in the 4-mg baricitinib group vs. the placebo group (−6.9 vs. −5.6 joints). However, the extent and severity of skin lesions (as assessed by the area and severity index of cutaneous lupus erythematosus) did not improve with baricitinib treatment compared to the placebo group. There were also no significant differences in the changes in anti-dsDNA antibodies and complement levels between the baricitinib and placebo groups.

Although the occurrence of adverse events was similar among the three groups in the trial (57), serious infections were more common in the 4 mg baricitinib group (6%) than in the 2-mg baricitinib group (2%) and placebo group (1%). One patient with SLE who was positive for antiphospholipid antibodies and treated with 4 mg baricitinib developed a deep vein thrombosis (accounting for 1% of patients treated with 4 mg baricitinib). Although the effect of baricitinib in reducing joint tenderness is very small, the results of this trial provided a positive signal for further phase III trials of JAK inhibitors for various symptoms of SLE.

Two multicenter, randomized, placebo-controlled phase III clinical trials of baricitinib in non-renal SLE are underway (NCT03616912 and NCT03616964). Solcitinib (GSK2586184), a selective JAK1 inhibitor, was going to be tested in a Phase II trial (NCT01777256) in patients with active non-renal SLE; the trial was stopped early after the recruitment of 50 patients, due to inadequate efficacy. No significant effect on the mean expression of IFN transcriptional biomarkers was observed (58). In addition, drug reactions exhibiting eosinophilia and systemic symptoms associated with solcitinib were observed in two patients (4%) and reversible hepatic dysfunction was documented in four patients (8%) (58, 59). More clinical data are needed to confirm the selective effects of selective JAK inhibitors and their efficacy and toxicity.

Based on the limited information available in the literature (57, 60), JAK inhibitors are expected to provide an alternative treatment option for patients with non-life threatening lupus who are refractory to standard therapeutic management, such as those with joint or skin disease. Many new JAK inhibitors are currently in development and will be tested in patients with SLE, and it is hoped that more-effective and less-toxic drugs will soon be available to continue to improve the prognosis of SLE patients.

### KIM-1 as a Urinary Biomarker in Lupus Nephritis

**Figure 2:**
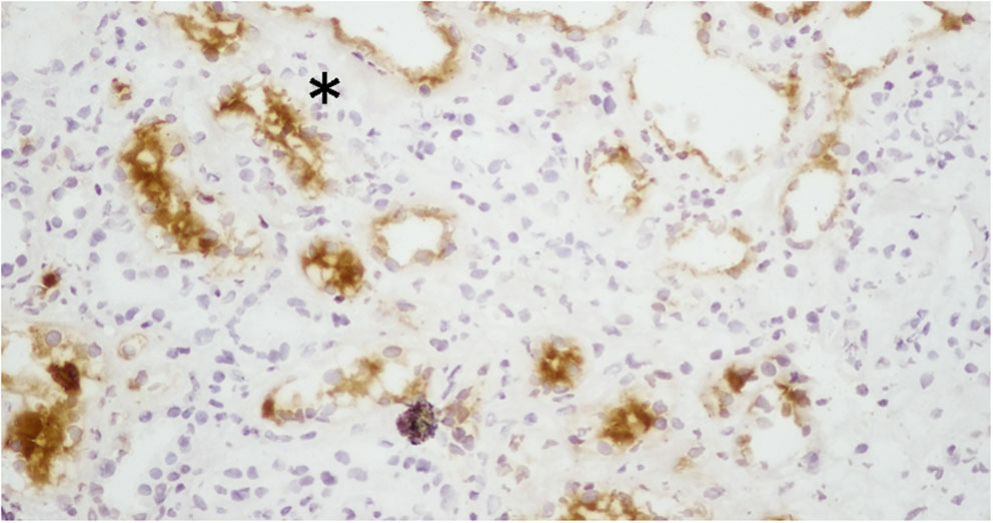
Expression of KIM-1 (kidney injury molecule-1) in the proximal tubules of patients with lupus nephritis. The expression of KIM-1 in this renal biopsy of a patient with LN was stained by immunohistochemistry. KIM-1 was not present in the glomeruli, but it was present in the damaged and dilated tubular cells. *The expression of KIM-1 is observed on the apical surface of proximal tubular cells in the kidney.

Kidney injury molecule-1 (KIM-1) and TIM-1, which are the same molecule, are relatively recently discovered transmembrane proteins with Ig-like and mucin domains in their ectodomain. TIM-1 modulates CD4^+^ T-cell responses and is also expressed by damaged proximal tubules in the kidney (where it is known as KIM-1). KIM-1 is upregulated more than any other protein in the proximal tubules of the kidneys and with various forms of injury (61, 62) (Figure 2). KIM-1 is a phosphatidylserine receptor that mediates the phagocytosis of apoptotic bodies and oxidized lipids (63). A chronic expression of KIM-1 leads to progressive renal fibrosis and chronic renal failure (64), which is speculated to be due to oxidized lipids; KIM-1 is associated with phagocytic functions that take up toxic substances such as oxidized lipids.

In addition to its role in phagocytosis, KIM-1 can activate signaling through the PI3K pathway (65). The role of KIM-1 signaling in proximal tubular cells and the link between KIM-1 phagocytosis and phosphorylation remain to be determined. Yang et al. observed that KIM-1-mediated phagocytosis functions downregulate the inflammation and innate immune responses in acute ischemic and toxic injury (66). It is thought that KIM-1 has a role in tubular interstitial damage (67). The expression of tubular KIM-1 is specific to ongoing tubular cell damage and de-differentiation (68, 69), and urinary concentrations of KIM-1 are thought to reflect this expression. KIM-1 is also associated with renal interstitial fibrosis and inflammation in certain types of renal disease (70).

Regarding prognostic factors, Austin et al. reported that tubular atrophy and fibrosis are associated with poor prognosis in LN (69). LN is often associated with comorbid acute and chronic pathological renal changes, and understanding the extent of renal damage without invasive testing is important in determining a patient's renal prognosis. The majority of tubular KIM-1 (~90%) in various human renal diseases is of proximal origin, as was identified by double-labeling studies with aquaporin-1 (a marker for proximal tubules) (71). KIM-1 is localized in the apical membrane of dilated tubules in acute and chronic tubular injury (72). The localization of KIM-1 expression appears to be related to the susceptibility of specific tubular segments to different types of injury (72). The selective KIM-1 expression by injured proximal tubular cells provides a strong impetus for using KIM-1 as a biomarker of damage.

Elevated urinary KIM-1 levels are strongly related to the tubular KIM-1 expression in experimental settings and in human renal disease (71, 72). We observed a significant correlation between urinary KIM-1 levels and the activity in LN by and enzyme-linked immunosorbent assay (ELISA) in humans and mice (61, 73). In the former study, we assessed the urinary KIM-1 level and tubular KIM-1 expression in kidney biopsies of SLE patients and their association with histological markers of renal damage (61), and we found that the urinary KIM-1 levels were significantly correlated with proteinuria (*R* = 0.39, *p* = 0.004) and with tubular damage (*R* = 0.31, *p* = 0.01). To assess the diagnostic value of urinary Kim-1 as a novel marker for crescent formation and interstitial infiltration, we used a receiver operating characteristic curve analysis to determine a cut-off level for urinary KIM-1 levels. At urinary KIM-1 levels >11.2 ng/day, the assay had 62.5% specificity and 100% sensitivity for the diagnosis in patients with cellular crescent formation. At urinary KIM-1 levels >3.2 ng/day, the assay had 60.8% specificity and 87.5% sensitivity for the diagnosis in patients with interstitial infiltration (61). Elevated urinary KIM-1 levels were strongly associated with tubular KIM-1 expression in both an experimental setting and human renal disease, and it was revealed that urinary KIM-1 is a very promising biomarker for the presence of tubular interstitial pathology and damage (61, 74, 75).

Several studies have shown that in patients with other forms of renal injury (including ischemia, inflammation, and nephrotoxic drug injury), the renal cortical and medullary expression of tubular KIM-1 in damaged tubules is up-regulated after the disease induction (74, 75). In clinical practice, it is essential to evaluate patients' kidney status. A renal biopsy is a standard diagnostic tool for the evaluation of kidney lesions in SLE, but due to its invasive nature, a kidney biopsy has potential risks and as a rule, it is not routinely performed. Moreover, a small amount of tissue may not be representative of the entire kidney (76). It is thus highly desirable to identify early and reliable biomarkers of kidney lesions in SLE (77, 78).

### Mechanisms Downstream of the TIM-1/TIM-4 Signaling Pathway

Different anti-TIM-1 antibodies that are specific to the IgV domain of TIM-1 have different effects on immune cell activation and response, due mainly to their different binding activities. A high-affinity anti-TIM-1 antibody, 3B3, forms a stable TIM-1 complex and brings TIM-1 into the TCR-CD3 complex, which enhances T-cell function and helps to form a large molecular activation cluster for complete T-cell activation (79). The low-affinity antibody RMT1-10 has an inhibitory effect and may not support the formation of a stable TIM-1-TCR-CD3 complex (80).

Foxp3-expressing regulatory T cells (Tregs) helped regulate the autoimmune response and provide protection against a murine model of LN (81). Treatment with the low-affinity antibody RMT1-10 increased the number of foxp3^+^ cells in the thoracic cavity and the percentage of foxp3^+^CD4^+^CD25^+^ cells in the spleen of the mice. RMT1-10 modulates the immune-response regulatory B cells (Bregs) and CD19^+^CD5^+^ cells, and IL-10-producing cells may be involved in the effects of TIM-1 by increasing the percentage of CD19^+^CD5^+^ and IL-10-producing cells. TIM-1 expression has been reported in activated T cells (82), DCs (83), and B cells (84). In association with this loss of IL-10 production in Bregs, the mice developed features of systemic autoimmune disease, including activated T cells with autoantibody formation and high IFN-γ production (85).

### The Blocking of TIM-1/TIM-4 Pathway Agents

**Figure 3:**
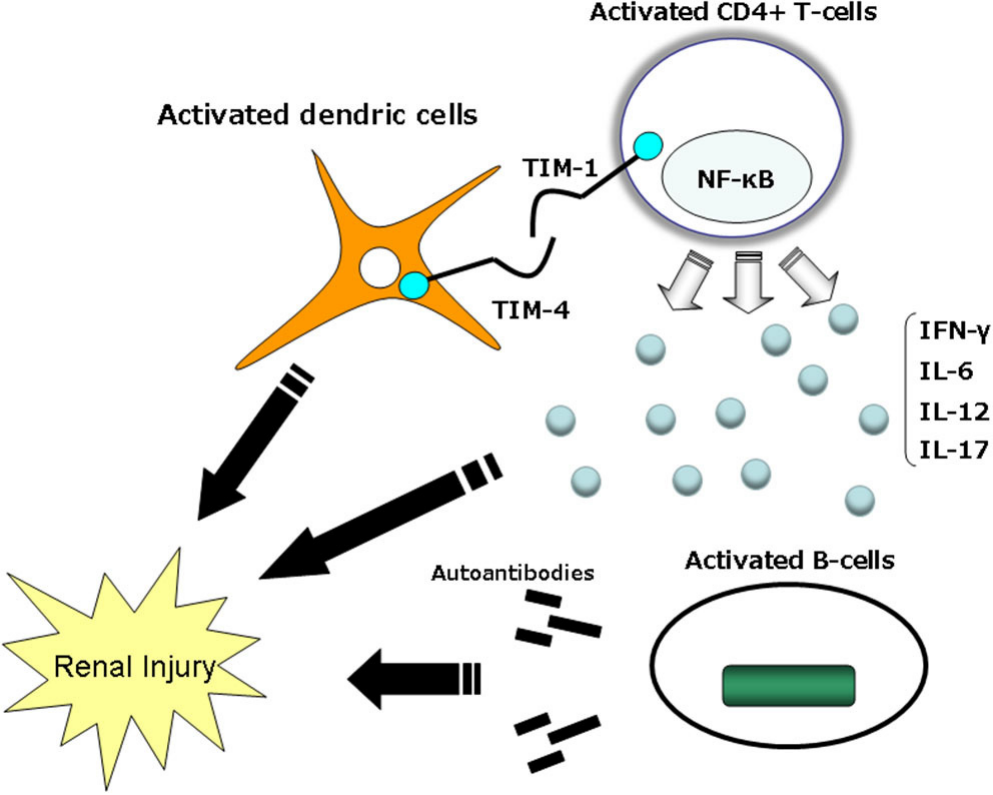
Mechanisms by which TIM-1–TIM-4 might modulate immune function. TIM-1 expression is observed to be higher on Th2 cells than Th1 cells. TIM-4 on the surface of mature dendric cells interacts with TIM-1 on the surface of activated T cells and delivers a stimulatory signal into the T cell, thus enhancing T-cell expansion and effector functions. TIM, T-cell immunoglobulin mucin domain.

Macrophages and CD4^+^, CD8^+^, and CD4-CD8-B220^+^ T cells are present in the kidneys of individuals with LN. Leukocyte recruitment is influenced by cytokines and chemokines, which correlate with the degree of tissue damage and predict disease progression (86, 87). Autoantibodies are important, and the T-cell population of T-cell-deficient MRL-Faslpr mice, which are prone to lupus, do not develop autoantibodies or immune complex disease (88–90). The tissue injury in LN is thus mediated by both autoantibodies and autoreactive lymphocytes (73, 91). TIM-1 can bind to TIM-4, which is expressed on antigen presenting cells (APCs) (83) (Figure 3). TIM-1-TIM-4 interactions on macrophages contribute to T-cell activation and macrophage-induced autoimmune nephritis (19, 81, 92). In the direct pathway, TIM-1 expressed on activated T cells cross-links with TIM-4 and directly activates macrophages.

TIM-4 is not expressed on T cells, but it is expressed on APCs, especially mature lymphoid DCs (92). TIM-4 binds to activated T cells expressing TIM-1, and TIM-1 binds to DCs expressing TIM-4; all of the fusion protein binding is mediated by TIM. It was demonstrated that RMT1-10 could be specifically blocked by a monoclonal antibody specific to TIM-1 (92).

The antibody RMT1-10 was shown to inhibit both Th1 and Th17 responses without a significant inhibitory effect on Th2 responses, Tregs or Bregs, as a low-activity antibody; treatment with RMT1-10, when administered after the development of autoimmunity and the progression of renal damage, suggesting that manipulation of TIM-1 may have potential therapeutic applications for LN. We have examined the low-affinity antibody RMT1-10 in experimental studies (73, 73, 74). In a murine lupus model, treatment with RMT1-10 attenuated the progression of lupus nephritis by prolonging survival and affecting a range of important mediators (73).

The renal manifestations of the systemic autoimmune disease SLE are characterized by the expression of autoantibodies in response to nuclear antigens, and they are associated with immune injury and a local inflammatory tissue response (93). Reduced autoantibody production is associated with a reduced recruitment of glomerular macrophages and reduced depositions of glomerular IgG and complement in brought about by RMT1-10. The serum anti-DNA antibody of IgG2a, whose switching is also known to be dependent on Th1 cytokines, was significantly reduced in RMT1-10-treated mice (94); circulating anti-DNA antibodies of IgG3 were associated with glomerulonephritis in MRL-Faslpr mice (73). These results suggest that an anti-TIM-1 antibody may affect not only the cytokine response, but also the ability to produce antibodies and immunoglobulins in LN.

## Conclusion

The mechanisms of JAK-STAT and TIM-1/TIM-4 signaling pathways in controlling the inflammatory network in LN have been briefly explained herein. The JAK-STAT pathway sends signals from extracellular ligands such as cytokines, chemokines, growth factors, and hormones directly to the cell nuclei. Because the JAK-STAT pathway plays a major activating role in a variety of disease processes, extensive efforts have been made to develop specific inhibitors of this pathway. The JAK-STAT pathway and its components have been used in immunology for the regulation of various diseases, and this pathway is a good candidate for targeted interventions. The increased evidence of JAK-STAT activation in the pathogenesis of renal injury establishes a new set of targets for potential interventions in this disease. The TIM-1/TIM-4 pathway contributes to pro-inflammatory cytokines and triggers T-cell activation and macrophage activation. In the direct pathway, TIM-1 on activated T cells cross-links with TIM-4 and directly activates macrophages. In the indirect pathway, TIM-1 on activated T cells triggers IFN-γ production, resulting in the activation of macrophages. TIM-1 plays an important role in the development of systemic autoimmunity and its effects on end organs. The low-affinity antibody RMT1-10 inhibits both Th1 and Th17 responses without having a significant inhibitory effect on Th2 responses, Tregs or Bregs. As a result, TIM-1 appears to have potential as a therapeutic agent for LN.

## Author Contributions

The author confirms being the sole contributor of this work and has approved it for publication.

## Conflict of Interest

The author declares that the research was conducted in the absence of any commercial or financial relationships that could be construed as a potential conflict of interest.
